# Ultra-Flexible and Large-Area Textile-Based Triboelectric Nanogenerators with a Sandpaper-Induced Surface Microstructure

**DOI:** 10.3390/ma11112120

**Published:** 2018-10-29

**Authors:** Jian Song, Libo Gao, Xiaoming Tao, Lixiao Li

**Affiliations:** 1 College of Civil Engineering, Shenzhen University, Shenzhen 518060, China; dfsongjian2006@126.com; 2 Nanotechnology Center of Functional and Intelligent Textiles and Apparel, Institute of Textiles and Clothing, The Hong Kong Polytechnic University, Hong Kong 999077, China; xiao-ming.tao@polyu.edu.hk; 3 Department of Mechanical and Biomedical Engineering, City University of Hong Kong, 83 Tat Chee Avenue, Kowloon, Hong Kong 999077, China; bogao5-c@my.cityu.edu.hk

**Keywords:** triboelectric nanogenerator, textile, ultra-flexible, large-scale, theoretical model

## Abstract

Wearable triboelectric nanogenerators (TENGs) have attracted interest in recent years, which demand highly flexible, scalable, and low-cost features. Here, we report an ultra-flexible, large-scale and textile-based TENG (T-TENG) for scavenging human motion energy. The triboelectric layer was derived from the polydimethylsiloxane (PDMS) film with a cost-effective paper-induced rough surface via a facile doctor-blending technology. Ag-coated chinlon fabric (ACF) with ultra-flexible, large-scale and conductive characteristics was used as the electrode. The as-fabricated PDMS-based ACF (PACF) composites possess a 240 × 300 mm^2^ superficial area and remain highly flexible under mechanical squeezing, folding and even tearing deformation. The maximum output charge of ~21 μC and voltage of 80.40 V were therefore achieved to directly power 100 LEDs based on the high surface area of 762.73 mm^2^ which was rationally replicated from the sandpaper of the T-TENG. Moreover, the output voltage signal can be also used as a trigger signal of a movement sensor. Importantly, the explicit theoretical model corresponding to T-TENG was quantitatively investigated under different applied force, frequency and effective surface factor.

## 1. Introduction

Micro/nano-scale energy scavenging has been taken into consideration as a promising green technology to generate energy from daily human motion, which can be expectedly integrated into wearable and self-powered systems [1,2,3]. Textile-based flexible electronic devices that are light and permeable can be made in very large areas of over hundreds or thousands of square meters [4,5]. Among them, textile-based triboelectric nanogenerators (T-TENGs), as excellent energy harvesters, have recently attracted great attention [6,7,8]. Table 1 summarized some fiber/textile-based triboelectric nanogenerators. As compared to flexible thin-film-based TENGs, textile-based TENGs have outstanding three-dimensional deformability, superior fatigue resistance in long-term repeated large deformation, and excellent ubiquitousness, perfectly suited for wearable electronics, human-machine interaction as well as large-area applications. Furthermore, the mass fabrication of textiles normally involves cost-effective and environmentally friendly processes by using well established facilities at low temperature, often in ambient conditions.

T-TENGs typically adopt organic polymeric materials, i.e., polydimethylsiloxane (PDMS), as triboelectric layers and conductive fiber yarns/textiles as electrodes following the triboelectric effect and electrostatic induction [9]. Apart from using contact materials with larger polarization variation, rationally constructing surface microstructures is another useful route to further improving the electric performances of T-TENGs by increasing the effective contact area [14,15,16,17,18,19]. Nowadays, creating surface micro/nanostructures on the contact layers of TENGs are mostly based on two strategies: surface etching and surface replication. For the former, a relative roughness contact surface can be obtained by etching polymer surface by nanoparticles. Lin et al. fabricated polymer nanowire structures on the contact surfaces via inductively coupled plasma (ICP) etching [18]. Lee et al. adopted a reactive ion etching (RIE) nanotechnology to create the nanostructured configuration of contact material surface, and a 4.6-time current enhancement based on the nanopatterned T-TENG were achieved compared to the T-TENG with a flat surface [3]. For the latter, by solidifying liquid-phased polymers in a mold with micro/nanocavities, or liquid phase-assisted technology, the surface micro/nanostructures can be achieved [20,21]. Currently, many surface microstructures, such as pyramids [22,23], cube [24] and nanorod array [25], have been investigated by copying the surface configurations of mold. Yang et al. designed pyramid-type surface patterning to fabricate surface structure by surface replication technique, and the output voltage of the resultant TENG was increased by 67% compared to that without structure architecture [19]. Fan et al. fabricated various PDMS pattern arrays (line, cube and pyramid) to enhance the friction effect. The result clearly illustrates that the output efficiency corresponding to the TENGs with various surface microstructures can be ordered as “film < line < cube < pyramid” [24]. However, multiple postprocesses, i.e., transferring/integrating the as-fabricated dielectric to the electrode, are essential for fabricating the TENG, giving rise to a multi-step technology [26]. Likewise, the cost of manufacture mold and surface micro-/nano-manufacture also increase the manufacturing costs. Therefore, the development of facile, inexpensive and one-step manufacture technology to generate microstructures on the contact surface in TENGs is strongly needed.

In this regard, we fabricated the textile-based triboelectric nanogenerators using commercially available knitted chinlon fabric coated by silver (Ag@CF, or ACF) and PDMS film by a one-step doctor-blading method. Here, silver fabric has antibacterial and radiation-proof properties, which is greatly beneficial to health [27,28]. PDMS film has been widely used as a promising multifunctional soft matter since it has many advantages, i.e., high flexibility and bio-compatibility [12,29]. Importantly, by adopting commercial sandpapers with different surface roughness, the microstructure arrays can be fabricated on the surface of PDMS film in a one-step process. After blading the PDMS on the surface of sandpaper by a glass rod, an ultra-flexible and large-scale ACF was immediately coated on the PDMS film. After drying for 30 min in the oven, a free-standing PDMS-based ACF (PACF) with a specific surface morphology was fabricated by directly peeling off from the sandpaper, which was assembled with another ACF to form the T-TENG for the first time. The influence of microholes on the electric performance of T-TENG was firstly investigated to find out the optimized T-TENG. Then, the corresponding electric performances, i.e., resistor match and charging behavior, based on the optimized T-TENG were explored. Furthermore, the practical application as a wearable harvester for scavenging mechanical energy and as a load sensor were exhibited. More meaningfully, the explicit theoretical model was established to quantitatively investigate the electric behaviors of T-TENG, which was fully verified by the corresponding experiment. On the basis of the established model, the influences of gaps between the two contact surfaces, frequency, surface charge density and effective surface factor on the electric performances of T-TENG were first predicted, which agree with the related experimental data. We expect such methods, including experiments and theoretical models, as well as our findings could be further beneficial to design a more facile, practical and low-cost T-TENGs in the flexible wearable device field.

## 2. Materials and Methods

### 2.1. Materials

The materials for T-TENG should meet the following requirements: (I) The electronegative polymer with high yield strength but low modulus is ranked relatively low in the triboelectric series, such as polydimethylsiloxane (PDMS) and polytetrafluoroethylene (PTFE) [30]. (II) All the materials are flexible, i.e., conductive fibric (CF) and polymer thin film. (III) The triboelectric surface possesses a specific microstructure or relatively roughness.

**Figure 1:**
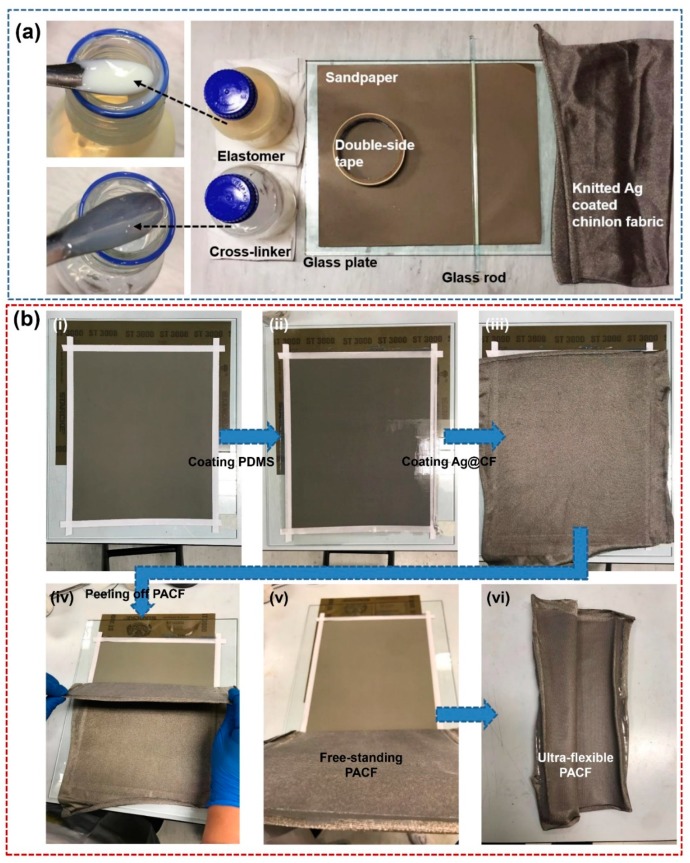
(**a**) Materials and fabrication equipment. (**b**) Schematic illustration of one-step fabrication process for PDMS-based Ag@CF composite film.

In this work, the elastomer PDMS and the related cross-linking agent adopted XE15-645 (Tokyo, Japan). Ultra-flexible, conductive and radiation-proof Ag@ chinlon fabric (ACF) was purchased from LuFeier^TM^ (Jiangsu, China). Low-cost sandpapers (Matador, Shanghai, Germany) were adopted to create a convex–concave surface microstructures. Figure 1a demonstrates the materials that were used to fabricate the T-TENGs. Here, ACF (electric resistance: ~0.1 Ω) is ultra-flexible, large-scale, artistic and conductive, thereby resulting in that it is suitable to be used as wearable electrodes. The Raman spectrum of polymer material is consistent with that of PDMS (Figure S3a, ESI). PDMS film also demonstrates the Young’s modulus of 1.59 GPa and yield strength of 0.4 MPa (Figure S3b, ESI), which is inexpensive and flexible. In addition, we adopted the low-cost sandpaper (~$0.0025 cm^−2^) as the substrate to fabricate the surface structure of PDMS film (Figure 1a).

### 2.2. Fabrication of PDMS-Based Ag@CF Composites and T-TENG

**Figure 2:**
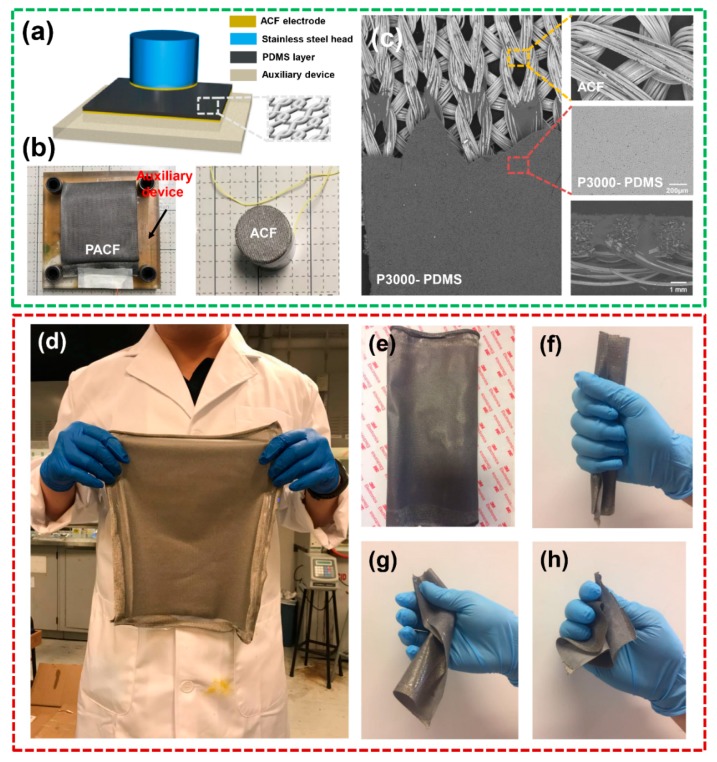
(**a**,**b**) Schematic and practical images of T-TENG assisted by an auxiliary device. (**c**) SEM image of PACF composite film, inset ACF electrode, surface microstructure of PDMS and cross-sectional SEM image of PACF. (**d**) Ultra-flexible and large-scale PACF composite film. (**e**–**h**) Images of PACF in cases of being compressed, warped and folded.

Firstly, PDMS (A) as well as cross-linking agent (B) were blended by 1:1 (mass ratio) to homogeneously synthesize the PDMS solution (Figure 1a). Then, a piece of sandpaper with various roughness was attached to a glass plate using double-sided tapes on the two sides of sandpaper (Figure 1b(i)). Here, the tapes have two effects: (1) Fixing the position of sandpaper on the glass plate. (2) Because the glass rod can only move along the protruding tapes, the resultant PDMS coated ACF composites possess different thickness via increasing/decreasing the layer number of tapes. Subsequently, the PDMS solution was casted at one end of the sandpaper, and PDMS film without being completely solidified was fabricated on the sandpaper surface by gradually gliding a glass rod (Figure 1b(ii)). Likewise, the ACF was immediately covered on the surface of PDMS film (Figure 1b(iii)). After curing at 80 °C for 0.5 h in an oven, the PACF composites with 280 × 220 mm^2^ were fabricated, which can be readily peeled off from the sandpaper substrate due to the weak interfacial properties between PDMS and sandpaper (Figure 1b(iv,v)). Finally, the as-fabricated PACF and another ACF were selected as the friction layer and conductive layer to assemble T-TENG (Figure 2a,b).

The merits of the composite film are: (I) it can be readily fabricated, and the scale of film is only restricted by the sandpaper/substrate (Figure 1b(i–iii)). (II) The coated PDMS film possesses a microporous surface induced by inexpensive sandpaper (Figure 2c). (III) More interestingly, the as-fabricated PACF can be readily peeled off from the sandpaper, exhibiting a free-standing characteristic of PACF (Figure 1b(vi)). (IV) The fabrication process is only one-step. Compared with the second-time conjunction approach, the resultant composites possess an excellent interfacial property (Figure 2c).

### 2.3. Material Characterization

The morphology and elemental composition were measured by a scanning electron microscope (SEM, Tescan VEGA3, Czech), Raman spectroscopy. The surface roughness of the as-fabricated PACF was observed via using a 3D surface profile (Zygo, Nexview, Middlefield, NY, USA).

### 2.4. Performance Measurement

A Keyboard Life Tester (ZXA03, Zhongli, Dongguan, China) was used to provide a continuous dynamic sinusoidal motion to evaluate the electric performances of as-fabricated T-TENG. Simultaneously, a DAQ equipment (Dewetron, Dewe-2600 DAQ system, Austria) monitored the force signal, and a Keisight DSO-X3014A oscilloscope (Keisight, USA) with a N2790A high voltage probe (8 MΩ internal resistance) recorded the output voltage vs. time curves (The corresponding setup and the measured circuit can be seen in Figures S2 and S3, ESI).

## 3. Results and Discussion

### 3.1. Characterization of the Ultra-Flexible and Large-Scale T-TENG

Figure 2a,b demonstrates the schematic and practical images of the T-TENG assisted by an auxiliary device. The PACF composite film with a surface area of 6 × 6 cm^2^ was fixed on one side of the auxiliary device, and a piece of ACF with a 762.73 mm^2^ surface area was adhered on the opposite side by using double-side tape. Here, the auxiliary device can be used to control the size of contact area and contact time in order to quantitatively elaborate the triboelectric performances of T-TENG.

Furthermore, the free-standing PDMS was uniformly coated on the surface of ACF, ensuring an excellent linking property between PDMS and conductive fabric (Figure 2c). Figure 2d shows that the as-fabricated PACF composite film can be readily compressed, warped and folded, indicating a potential application to be incorporated into a wearable device.

**Figure 3:**
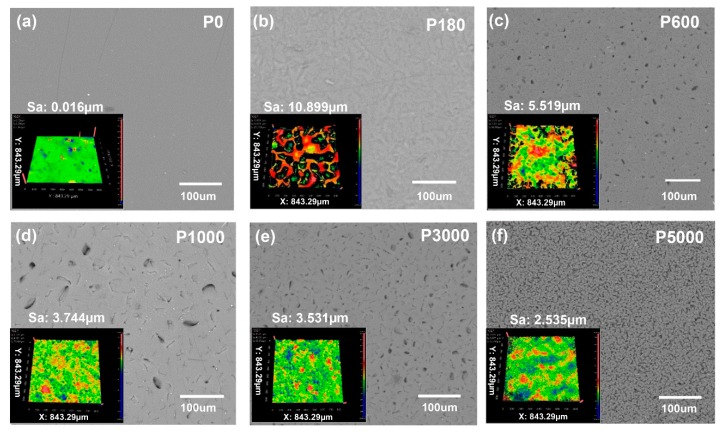
SEM morphologies of PDMS composites with different surface roughness. Insets illustrate the corresponding surface roughness. (**a**–**f**) The roughness of sandpapers corresponds to P0, P180, P600, P1000, P3000 and P5000, respectively. Note: “Sa” is average surface roughness.

Figure 3 shows the surface microstructures of the as-fabricated PACF composite film. The P0-PACF film has a relatively uniform surface, whereas the others demonstrate rough surface morphologies induced by sandpapers with various roughness degrees. Compared with these roughness surfaces, with the increase of the mesh of sandpapers, the surface of PACF composite film obviously changes to be smoother and the roughness decreases from 10.899 μm to 2.635 μm. This finding can to some extent increases the contact area, thereby improving the electric performances of T-TENGs.

### 3.2. Operation Mechanism of T-TENG

**Figure 4:**
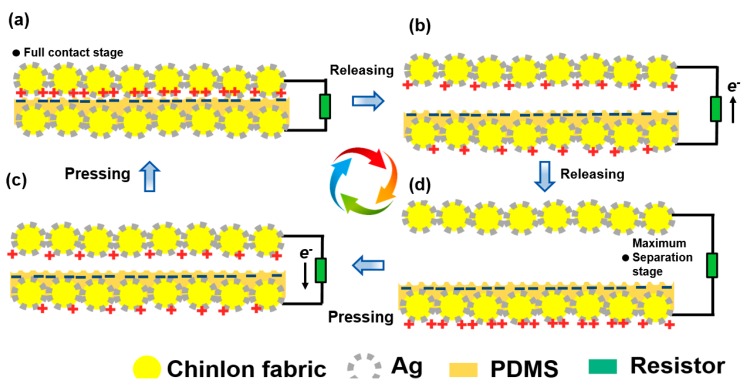
Working operation of T-TENG. (**a**) Full contact stage. (**b**) Releasing process from the full contact stage to maximum separation stage. (**c**) Maximum separation stage. (**d**) Pressing process from the maximum separation stage to full contact stage.

Figure 4 shows the schematic illustration of the operating mechanism of T-TENG in the case of contact-separation mode. Initially, taking into account triboelectrification, the ACF and PDMS films are brought into contact with each other by an externally applied load, giving rise to the occurrence of surface charge transfer at the contact area. In accordance with the triboelectric series [30], negative charges at the PDMS surface and positive ones at the ACF are generated (Figure 4a). When the T-TENG starts to be released, a potential difference induced by electrostatic effect is subsequently established between the two electrodes, where the potential difference can be calculated by

(1) $$U=\sigma \frac{\Delta d}{\varepsilon_{0}}\;$$

where *σ* means the surface charges density; *ε*_0_ is the vacuum permittivity; Δ*d* represents the gap distance. Likewise, due to the connection between the two electrodes, electrons flow from the bottom electrode to the top one to balance the potential difference, giving rise to an instantaneous current (Figure 4b). This is actually an electrostatic effect. When the upper electrode reaches the maximum separate distance, there is no dynamic electrode owing to the saturation state in two electrodes (Figure 4c). During the redirection pressure process, the accumulated electrons on the upper electrode flow back to the bottom electrode, and an opposite electric signal is obtained (Figure 4d). Consequently, an alternating current electric signal is generated by iterative contact-separate movement.

**Figure 5:**
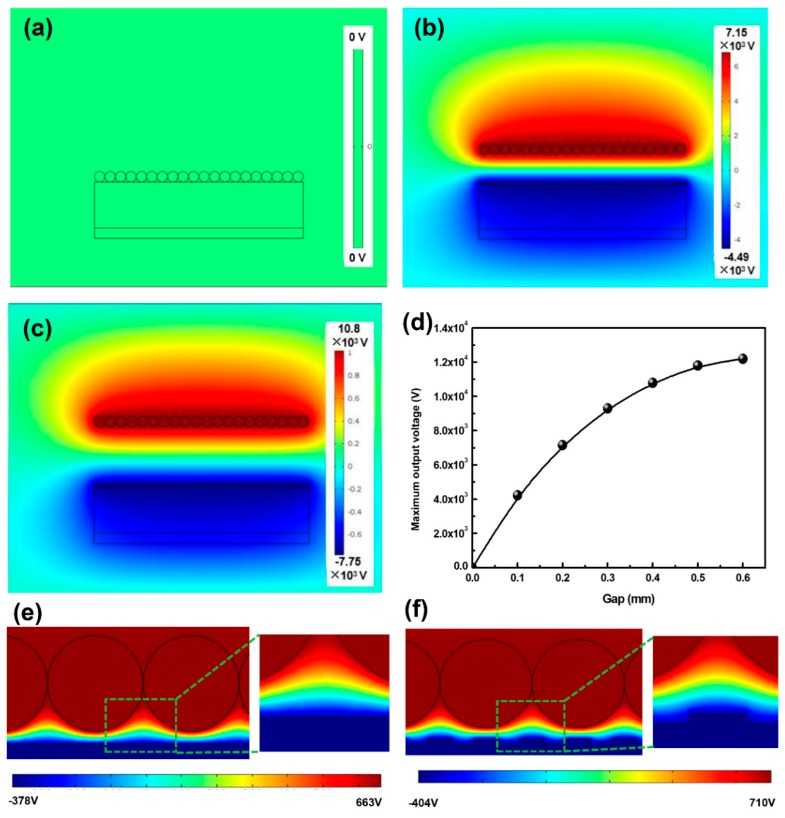
(**a**–**c**) Finite element simulation of the potential distribution in the T-TENG for different states. (**d**) The simulated relationship between the gap and maximum output voltage. (**e**,**f**) Potential distributions of T-TENG with the flat and sandpaper-induced surficial structures.

Finite element simulation was carried out to quantitatively investigate the potential distribution of T-TENG by using COMSOL software^©^ (COMSOL, Stockholm, Sweden), where a 2-dimensional (2D) model was established in COMSOL. Additionally, the circle-shaped structures represent the ACF, and microstructures induced by sandpaper used a set of rectangles for simulation. Figure 5a–c exhibits the simulation results with respect to the potential distribution with a gap between the ACF and PDMS of 0.4 mm, 0.2 mm and 0 mm, respectively. In the case of full contact status, the potentials on both PACF and ACF are completely equal to zero. The potential difference significantly rises with the increase of gap from 0 to 0.4 mm, which is consistent with the trend given in Equation (1). However, the relationship between the gap and maximum output voltage gradually approaches being nonlinear (Figure 5d), which can be ascribed to the parallel plate being unavailable when the gap exceeds the threshold.

In addition, Figure 5e,f illustrates the magnified potential distribution of T-TENG with the flat and sandpaper-induced surficial structures, where the gap is 0.01 mm. Compared with the potential distribution, the maximum output voltage of T-TENG with the microstructures is larger than that without the microstructures, which can be attributed to the larger contact surface for the T-TENG with the roughness surface.

### 3.3. Electric Performances of T-TENG

**Figure 6:**
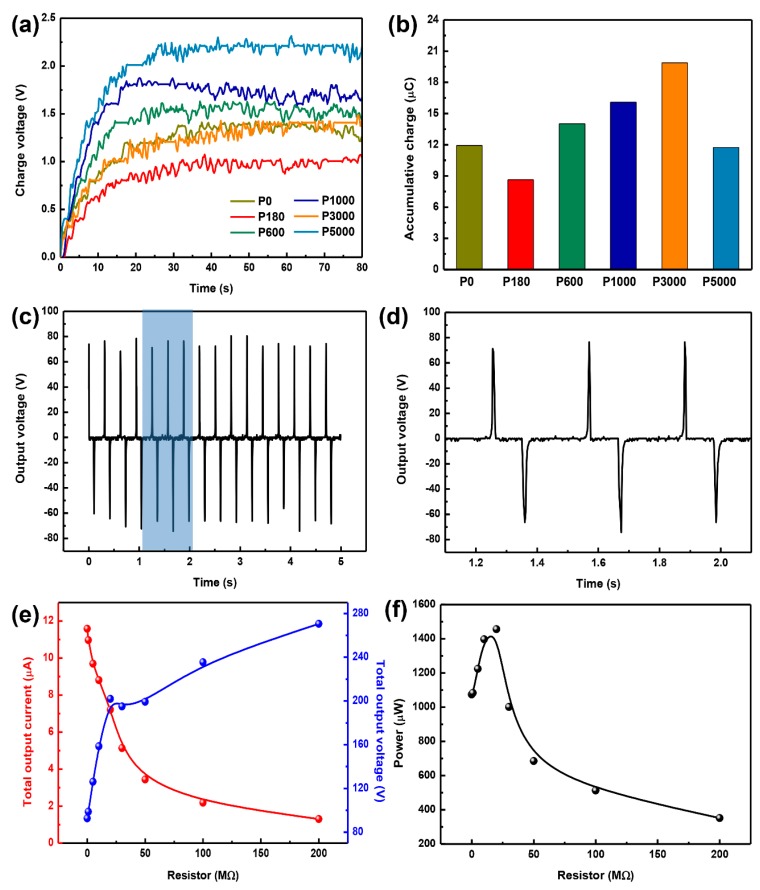
(**a**,**b**) Charging curves for 1 μF capacitor charged by T-TENGs with various surface roughness and the corresponding accumulative charge. (**c**) Output voltage vs. time curve. (**d**) Magnified image of voltage vs. time curve. (**e**) Total output current and voltage with varying load resistors. (**f**) Power output as a function of varying resistors.

To find out the optimized electric output electric performances of T-TENG with various surface roughness, P0-PACF, P180-PACF, P1000-PACF, P3000-PACF and P5000-PACF films were assembled into T-TENG with another ACF electrode, where P* designates the roughness class of the used sandpaper. The electric performances of as-fabricated T-TENGs were explored by charging a 1 μF capacitor under a 700 N compression load with a frequency of 3.24 Hz (The photograph of the measured equipment can be seen in Figure S1, ESI). It can be seen from the Figure 6a,b, that the amounts of charge within 80 s triggered by P0-T-TENG, P180-T-TENG, P1000-T-TENG, P3000-T-TENG and P5000-T-TENG are 11.93 μC, 8.63 μC, 14.01 μC, 16.08 μC, 19.87 μC and 11.74 μC, respectively. No obvious relationship between electric performance and roughness is obtained. More specifically, owing to the difference of surface morphologies induced by sandpaper (Figure 3), with the increase of sandpaper roughness from P180 to P3000, the effective contact surface can be to some degree enlarged, resulting in the improvement of electric performance. However, excess microholes can severely lessen the effective contact area of PDMS film, which leads to the decrease of electric performance. Additionally, an improved electric performance based on P3000-T-TENG, ~1.67 larger than that of P0-T-TENG, was achieved (Figure 6b).

Based on the optimized P3000-T-TENG with a 762.73-mm^2^ contact area, the output voltage as a function of time is shown in Figure 6c,d, in which the measured circuit is based on the circuit with an external resistor of 8 MΩ. The T-TENG can output a peak voltage of ~80 V and current of ~8 μA with an internal resistor of 8 MΩ. Due to the non-uniform contact surface, some small signals can be observed without compression load, which also has a contribution to increase the amount of transferred charge (Figure 6d). In addition, Figure 6e,f demonstrates the resistance dependence via adopting an external resistor varying from 0.1 MΩ to 200 MΩ, and the corresponding electric circuit is shown in Figure S2, ESI. As the increase of resistance, the current amplitude declines and the measured voltage with respect to internal resistor (8 MΩ) rises (Figure 6e). The instantaneous power shows a maximum value of 1.455 mW at the external resistance of 20 MΩ.

### 3.4. Application of the T-TENG Based on the Optimized P3000-PACF Film

**Figure 7:**
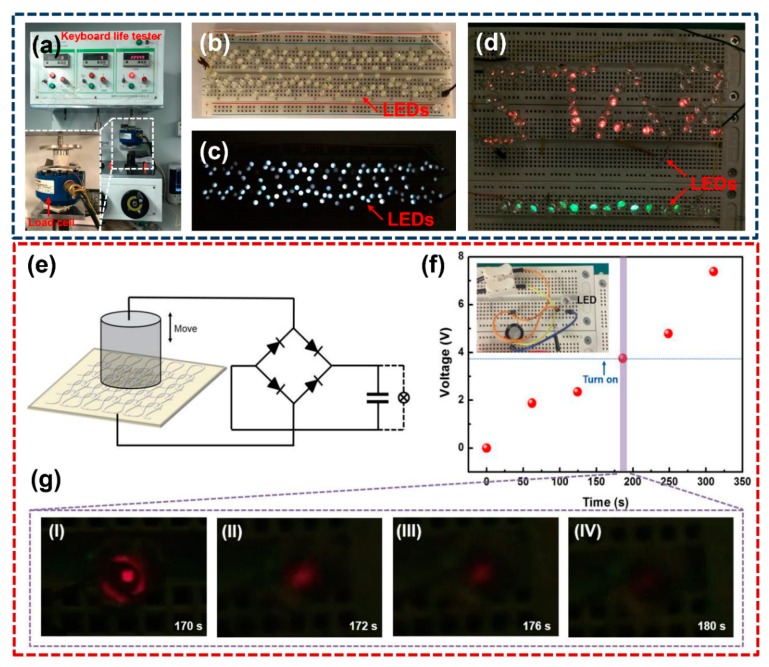
(**a**) Keyboard life tester (ZXA-03), inset the magnified image of cyclic compression equipment and P3000-T-TENG with a 762.73 mm^2^ contact area. (**b**,**c**) Photos of 120 LEDs together directly powered by the P3000-T-TENG. (**d**) A “STAR” shaped pattern directly powered by the P3000-T-TENG. (**e**–**g**) Electrical capacitive load characteristics of P3000-T-TENG and discharging process after shutting down the tester.

To exhibit the applications based on the P3000-T-TENG as a harvester, we employed the P3000-T-TENG with 762.73 mm^2^ contact area on the tester, which can generate a cyclic compression load (Figure 7a). Figure 7b–d demonstrates the P3000-T-TENG can directly power of 100 LEDs and light a “STAR” logo. Moreover, a 7.5 V output voltage can be obtained after charging a 47 μF capacitor for 350 s, and a LED can be lit for 10 s after turning on the switch at the time of 170 s (Figure 7f,g).

### 3.5. Electromechanical Responses of P3000-T-TENG

**Figure 8:**
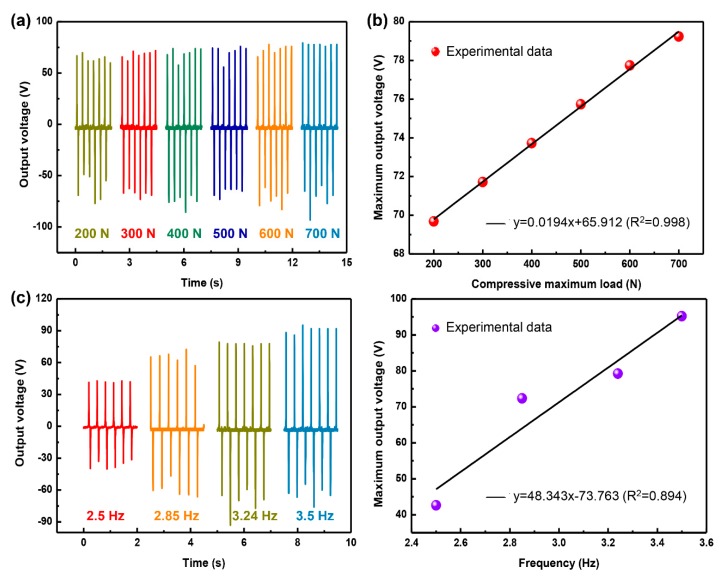
(**a**) Output voltage vs. time curves under various compressive loads. (**b**) Fitting curve of maximum output voltage as a function of compressive load. (**c**) Output voltage vs. time curves under various frequencies. (**d**) Fitting curve of maximum output voltage as a function of frequency.

Electromechanical responses of T-TENG (normal contact area: 762.73 mm^2^) with the P3000-PACF film were further tested to investigate the potential application as force sensor. The output voltage response under the frequency of 3.24 Hz as functions of external load is shown in Figure 8a,b, where the voltage increases linearly with the increasing load, demonstrating the T-TENG possesses an excellent advantage as the force sensor. This is mainly ascribed to the concave–convex structure within the contact surfaces of PDMS and ACF, resulting in a larger contact area under a stronger load (Figure 8b). Figure 8c,d also exhibits the influence of frequency on the output voltage of the same T-TENG under a load of 700 N. The maximum output voltage rises from 42.613 V to 95.204 V, with an excellent sensitivity of about 48.343 V Hz^−1^, but the linear relationship between maximum output voltage and frequency is not very well (R^2^ is only 0.894, Figure 8d), which could be attributed to the resilient time under various frequency is different.

**Figure 9:**
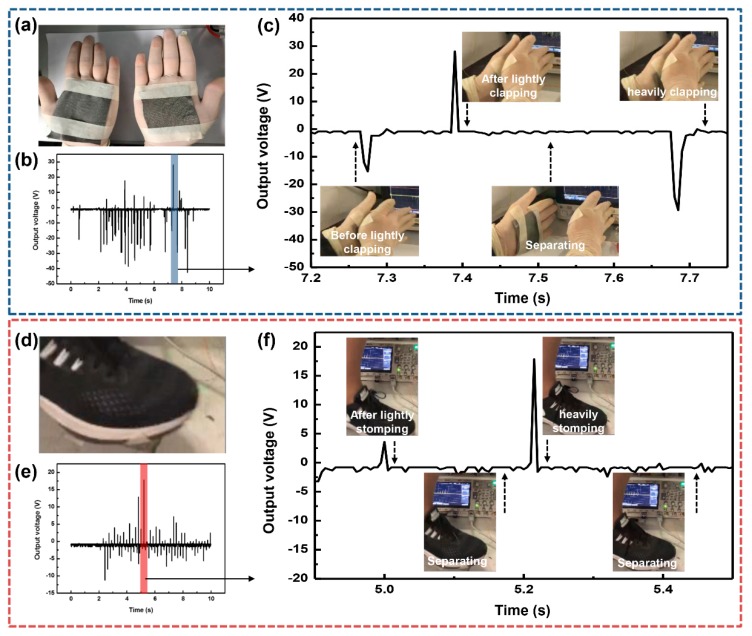
Illustration of the P3000-T-TENG harvesting energy from human motion. (**a**) Photograph of rectangle-shaped P3000-PACF composite film and ACF electrode taped to two palms, respectively. (**b**) Practically measured voltage in response to clapping palms under different force and frequency. (**c**) The corresponding status of clapping palms. (**d**) Photograph of P3000-PACF composite film and ACF taped to a shoe and floor, respectively. (**e**,**f**) Practically measured voltage in response to stomping and the corresponding status of a foot under repeated stomping. Note: the areas of P3000-PACF composite film and ACF are both 50 × 60 mm^2^.

Due to fact that T-TENGs are sensitive to external force as shown in Figure 8, the practical applications as an electromechanical sensor of T-TENG are exhibited in Figure 9. Figure 9a,b illustrates the output voltage of P3000-T-TENG driven by hand clapping at various loads and frequencies, which shows that the generated output voltage is strongly relevant with load and frequency. In other words, the larger load was implemented during clapping, the higher peak output voltage was generated. When the clapping frequency increases, relatively dense responses can be observed in Figure 9b. A magnified load-unloading process can be seen in Figure 9c, demonstrating the common clapping cases that completely correspond to the generated voltage responses. Note: the whole process in terms of clapping behavior can be found in Video 1, ESI.

The P3000-T-TENG was also assembled into a shoe to investigate the output signal generated by stomping behavior (Figure 9d–f). When stomping occurred, the P3000-T-TENG was subjected to continuously compressive load as illustrated in Figure 9d,e. Similarly, the heavy stomping behavior leads to higher peak voltage than the light one, and the generated voltage fully correspond to the loading-unloading cycles of the stomping behavior (Figure 9f). Note: the whole process in term of stomping behavior can be found in Video 2, ESI.

## 4. Theoretical Analysis and Verification for the T-TENG

In addition to exploring the electric performances via experiment and simulation, explicit theoretical model based on the above-mentioned analysis is an effective route to quantitatively investigate and predict the related performances of T-TENG.

**Figure 10:**
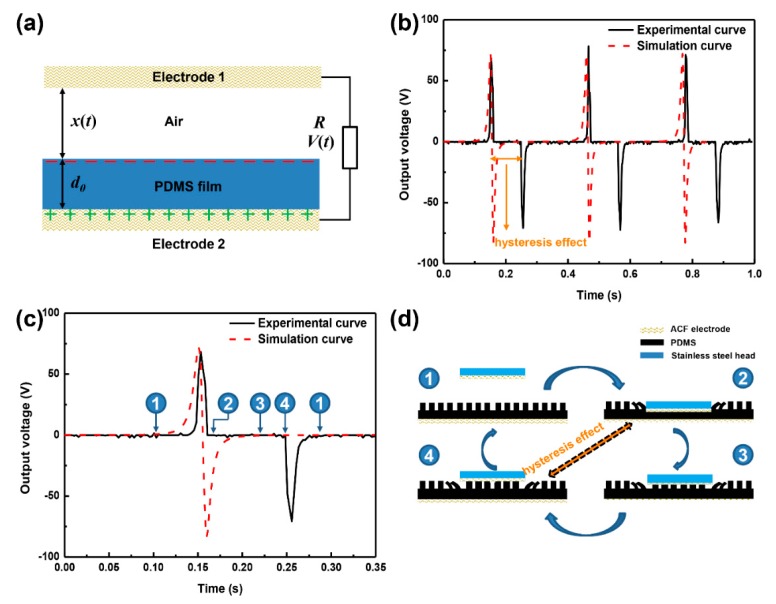
Comparison of experimental and theoretical prediction; (**a**) Output voltage vs. time curves. (**b**) Rectified output voltage vs. time curves. (**c**) Magnified image of voltage vs. time curve in one loading-unloading cycle. (**d**) Illustration of the corresponding reason of hysteresis effect.

Taking into account a conductor-to-dielectric TENG in case of contact-separation mode shown in Figure 10, we assume that the two triboelectric layers have equal density, *σ*, with the opposite polarity, after experiencing enough cycles [31]. When electrode 1 (ACF) reaches the maximum gap, *X*_max_, tribo-charges are fully located on electrode 2, leading to an equal amount of negative charges on the surface of the PDMS film (dielectric layer). This case is defined as the initial time, viz. *t* = 0, we can obtain

(2) $$\left\{ \begin{array}{l} Q_{1}(t=0)=0\\ Q_{2}(t=0)=\sigma S=Q \end{array}\right.\;$$

where *Q*_1_, *Q*_2_ are the amount of charge on electrodes 1 and 2. *Q* is the amount of transferred charges between electrodes 1 and 2 at the moment of *t* = 0. *S* is effective conduct area, viz. *S* = *ηS*_0_. Here, *S*_0_, *η* are the normal contact area and effective surface factor.

When the gap is reduced to *x*, based on the Gauss’s law, we can obtain

(3) $$E_{a}=\frac{Q_{1}}{S\varepsilon_{0}}\;$$

(4) $$E_{b}=-\frac{Q_{2}}{S\varepsilon_{0}\varepsilon_{r}}\;$$

where *E_a_*, *E_b_* are the electric field strength in the air gap and dielectric layer, respectively. *ε*_0_, *ε_r_* are the vacuum permittivity and relative permittivity.

Considering the close loop in circuit, we can obtain

(5) $$\sum_{i=1}^{N}V_{i}=0\;$$

Substitute Equations (3) and (4) into Equation (5), we can obtain

(6) $$E_{a}x(t)+E_{b}d+V(t)=0\;$$

where *x*(*t*), *d* are the air gap and thickness of dielectric. *V*(*t*) is the voltage of TENG at the time of *t*. According to the Ohm’s law *V*(*t*) can be expressed as

(7) $$V(t)=RI(t)=R\frac{dQ}{dt}\;$$

where *R* is the external resistor.

Substitute the Equation (6) into Equation (7), we can obtain

(8) $$R\frac{dQ}{dt}+\left(d_{r}+x\left(t\right)\right)\frac{Q}{S\varepsilon_{0}}-\frac{\sigma }{\varepsilon_{0}}x\left(t\right)=0\;$$

where *d_r_* represents the effective thickness of PDMS film (*d_r_* = *d*/*ε_r_*). By substituting the initial conditions given in Equation (2), Equation (8) can be explicitly solved as

(9) $$Q\left(t\right)=S\sigma -\exp\left(-\int_{0}^{t}\frac{d_{r}+x\left(\tau \right)}{RS\varepsilon_{0}}d\tau \right)\left(S\sigma +\int_{0}^{t}\frac{\sigma x\left(\tau \right)}{R\varepsilon_{0}}\exp\left(\int_{0}^{\tau }\frac{d_{r}+x\left(\gamma \right)}{RS\varepsilon_{0}}d\gamma \right)d\tau \right)\;$$

(10) $$\begin{array}{l} V\left(t\right)=R\frac{dQ\left(t\right)}{dt}\\ =-\frac{\sigma x\left(t\right)}{R\varepsilon_{0}}+\frac{d_{r}+x\left(t\right)}{RS\varepsilon_{0}}\exp\left(-\int_{0}^{t}\frac{d_{r}+x\left(\tau \right)}{RS\varepsilon_{0}}d\tau \right)\cdot \left(S\sigma +\int_{0}^{t}\frac{\sigma x\left(\tau \right)}{R\varepsilon_{0}}\exp\left(\int_{0}^{\tau }\frac{d_{r}+x\left(\gamma \right)}{RS\varepsilon_{0}}d\gamma \right)d\tau \right) \end{array}$$

Additionally, the movement equation of electrode 1, namely *x*(*t*), can be assumed as

(11) x(t)=Asin(ωt+φ)+A 

Table 2 listed all the experimental parameters used in solving Equation (10), and by using Mathematica software^©^ (Wolfram, Somerville, MA, USA), the explicit output voltage vs. time curves can be obtained.

Figure 10 demonstrates the comparison results of the (rectified) output voltage–time curve obtained by experiment and theoretical models in Equation (2). The predicted charge trend as well as maximum value agree with the corresponding experimental ones, while there is an obvious difference in response time of second-half impulse signal within a load–unload cycle. The hysteresis effect in the experimental voltage–time curve could be ascribed to two aspects: (i) the local deformation of surface microstructure; (ii) the PDMS film and ACF possess large deformation capacity (Figure 10d).

To be more specific, due to the electrostatic effect, electrons are driven from one electrode to another electrode, resulting in an instantaneous current (“①–②”, Figure 10c). When the external force is gone, the existence of convex–concave microstructures on the surface of PDMS, resulting in the occurrence of some small signals and hysteresis effect until to full separation of ACF electrode and PDMS film (“②–④”, Figure 10c). Afterwards, electrodes flow reversely to balance the potential difference, giving rise to an reverse instantaneous current (“④–①”, Figure 10c).

Moreover, Figure 10b illustrates the rectified voltage vs. time curves, and the amount of transferred charge in a loading-unloading cycle can be calculated by

(12) $$Q=\int_{0}^{T}Idt=\frac{1}{R_{i}}\int_{0}^{T}Udt\;$$

where *Q* is the amount of charge; *I*, *R_i_* and *U* means the current, voltage and internal resistance (8 MΩ); *T* is the period. In accordance with the Equation (2), the amounts of transferred charge obtained by experimental and theoretical voltage-time curves are 0.17 μC and 0.22 μC, and the relative error is ~29%, suggesting that the established theoretical model to some degree predicts the electric behavior of our TENG.

**Figure 11:**
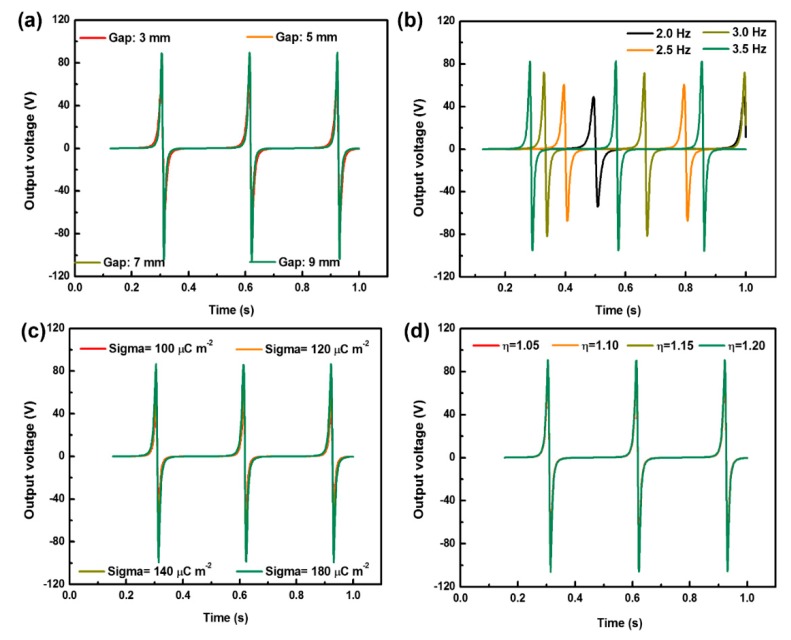
Influences of gap (**a**), frequency (**b**), surface charge (**c**) and effective surface factor (**d**) on output voltage behaviors.

**Figure 12:**
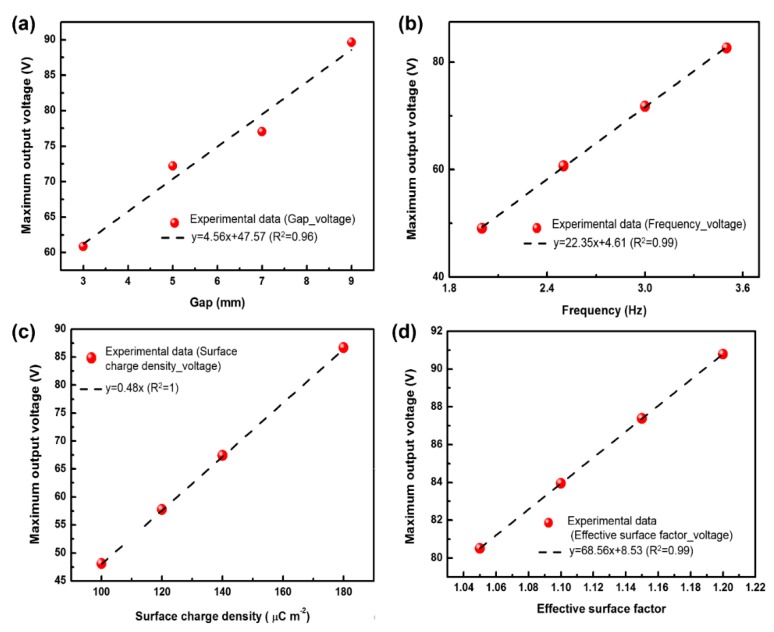
Relationships between maximum output voltage and gap (**a**), frequency (**b**), surface charge density (**c**) and effective surface factor (**d**).

Figure 11 shows the influences of various parameters, viz. gap, frequency, surface charge and effective surface factor, on the output voltage behaviors calculated by using the explicit control Equation (10), and the corresponding variation regulations are shown in Figure 12. With the increase of gap, frequency, surface charge and effective surface factor, the relevant maximum voltage linearly rises. More specifically, a higher output voltage can be obtained by taking into account the loading–unloading frequency (Figure 8b), which mostly agrees with the corresponding experimental result shown in Figure 8c,d. Owing to the approximately linear relationship between the maximum output voltage and effective surface factor, when the microstructures increase the contact surface area, the output voltage will rise. Nevertheless, in accordance with Figure 6a,b, once the surface roughness of sandpaper increases to 2.535 (P3000-T-TENG), a decline trend in output electric performance was found, which could be attributed that too many microstructures lead to the reduction of the contact area. These findings also support the validity of derived Equation (10). We hope this explicit equation can be used to further predict the triboelectric behaviors of other nanogenerators and find out the optimized match relationship between materials and structure.

## 5. Conclusions

In summary, assisted with the facile doctor-blending technology, a large-scale, ultra-flexible and free-standing PDMS-based Ag@CF with surface microstructures have been developed by a one-step method. The resultant PACF film was successfully used as a triboelectric layer, which can be readily assembled into the T-TENG with another electrode to scavenge mechanical energy. Compared to a P0-T-TENG, ~1.67-times improvement for electric performance based on P3000-T-TENG was achieved, and a maximum value of 1.455 mW for the instantaneous power at the load resistance of 20 MΩ was obtained. Based on the P3000-T-TENG, 100 LEDs can be directly driven, and a 7.5-V output voltage can be achieved after charging a 47-μF capacitor for 350 s, directly lightening a LED for 10 s. Apart from being a harvester, due to the electric behaviors being sensitive to load and frequency, this T-TENG can be also used as a load sensor. More specifically, an explicit theoretical model based on the principle of triboelectric effect was successfully built, which can be verified by the experimental output voltage vs. time curve. In accordance with the model, the influences of gap distance, frequency, surface charge density and effective surface factor on the electric performances have been quantitatively discussed.

## Figures and Tables

**Table 1:** Summary of fiber/textile-based triboelectric nanogenerators.

Type of Electrodes	Deposition Method	Area of the Surface	Maximum Load	Maximum Open Voltage	Maximum Power Density
Fiber-based TENG [9]	Coating	40 × 40 mm^2^	~11 N	150 V	~85 mW m^−2^
All-fiber TENG [10]	Electrospinning	60 × 50 mm^2^	-	210 V	700 mW m^−2^
Single fiber-based TENG [11]	Chemical grow	π × (22)^2^ μm^2^	-	7 mV	-
Textile-based TENG [12]	Coating	60 × 25 mm^2^	34–39 N	23.39 V	~1 mW m^−2^
Textile-based TENG [3]	Hot pressure	70 × 70 mm^2^	100 mm s^−1^	368 V	33.6 × 10^4^ mW m^−2^
Textile-based TENG [13]	Coating	100 × 100 mm^2^	-	40 V	-
Textile-based TENG [4]	Coating	50 × 50 mm^2^	-	50 V	393.7 mW m^−2^
Textile-based TENG [1]	Laser-scribing making	50 × 65 mm^2^	0.5 m s^−1^	120 V	1900 mW m^−2^

**Table 2:** Theoretical analysis parameters.

Symbol	Value
Internal resistor, *R*	8 (MΩ)
Normal contact area, *S*_0_	762.73 (mm^2^)
Effective surface factor, *η*	1
Thickness of PDMS film, *d*_0_	200 (mm)
Vacuum permittivity, *ε*_0_	8.854 × 10^−12^ (F m^−1^)
Relative permittivity of PDMS film, *ε_r_*	3.4
Amplitude of the movement equation (gap), *A*	5 (mm)
Initial phase angle, *φ*	π/2 (rad)
Angle velocity, *ω*	2π*f* (rad s^−1^)
Frequency, *f*	3.24 (Hz)
Initial surface charges density, *σ*	160 (μC m^−2^)

## Supplementary Materials

The following are available online at http://www.mdpi.com/1996-1944/11/11/2120/s1, Figure S1: Experimental setup. (**a**) Keyboard life tester (ZXA-03). (**b**) Magnified image of cyclic compression element and T-TENG. (**c**) Keisight DSO-X3014A oscilloscope and N2790A high voltage probe with 8 MΩ internal resistance. (**d**) Load supervisor system (Dewe-2600 DAQ system), Figure S2: Schematic illustration of measurement circuit. Note: the output voltage is not entirely the case of open-circuit or short-circuit, owing to the existence of an 8 MΩ (R2) built-in resistor, Figure S3: (**a**) Raman spectrum of PDMS film. (**b**) Stress vs. strain curve, Video S1: Video of repeated clapping palms under different force and frequency, Video S2: Video of repeated stomping under different force and frequency.
