# Supplementary material for: Ultra-Flexible and Large-Area Textile-Based Triboelectric Nanogenerators with a Sandpaper-Induced Surface Microstructure
Source: Materials (Basel). 2018 Oct 29;11(11):2120. doi: 10.3390/ma11112120 (PMC6266209; doi:10.3390/ma11112120)
Supplement: Supplementary file 1 [file materials-11-02120-s001.pdf]

materials

Supplementary information

Ultra-Flexible and Large-Area Textile-Based Triboelectric Nanogenerators with a Sandpaper-Induced Surface Microstructure

Jian Song [1,2,3,†], Libo Gao[3,†], Xiaoming Tao [2] and Lixiao Li [1,*]

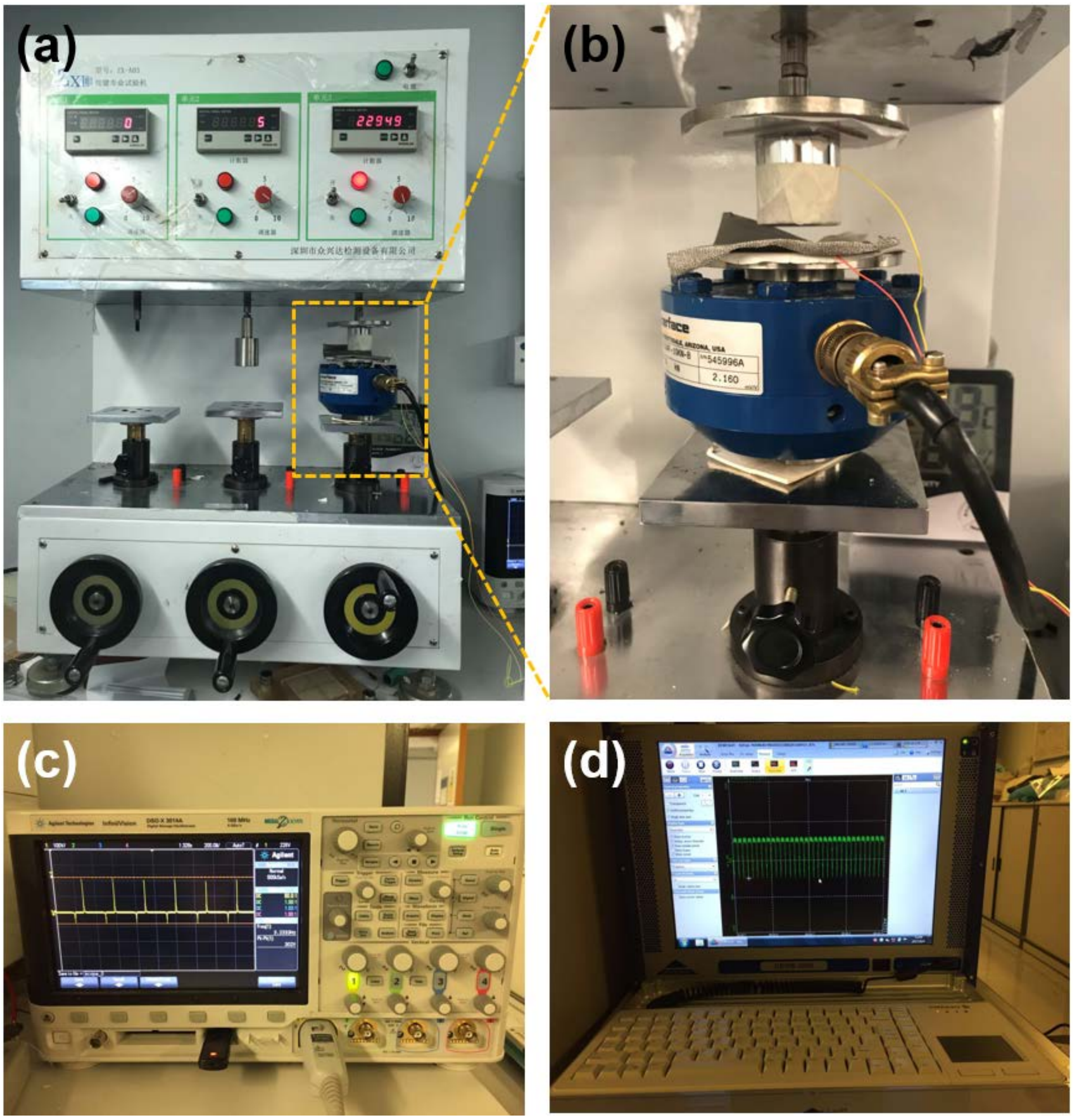

Figure S1. Experimental setup. (**a**) Keyboard life tester (ZXA-03). (**b**) Magnified image of cyclic compression element and T-TENG. (**c**) Keisight DSO-X3014A oscilloscope and N2790A high voltage probe with 8 MΩ internal resistance. (**d**) Load supervisor system (Dewe-2600 DAQ system).

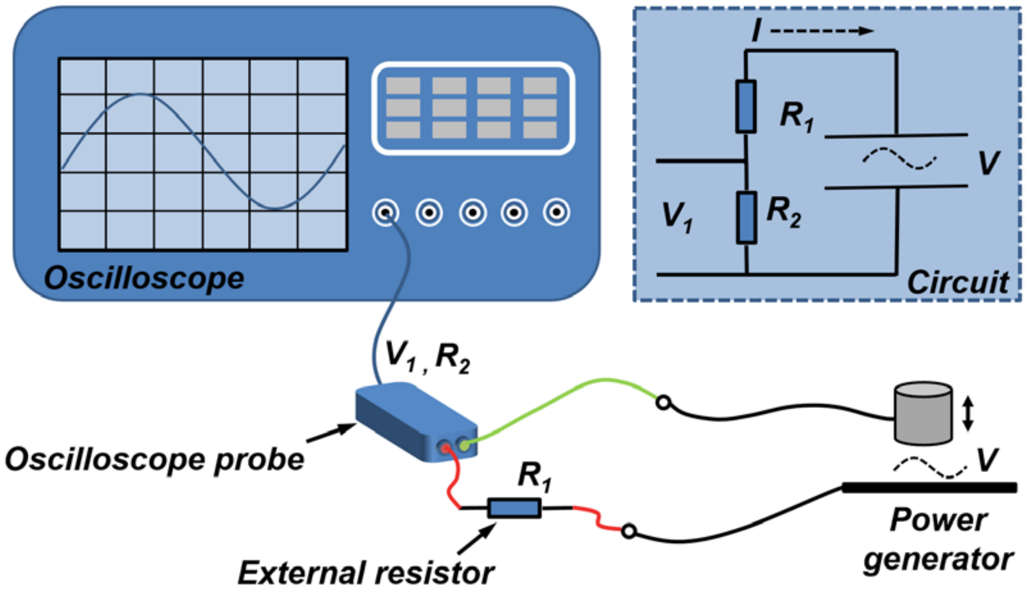

Figure S2. Schematic illustration of measurement circuit. Note: the output voltage is not entirely the case of open-circuit or short-circuit, owing to the existence of an 8 MΩ (R2) built-in resistor.

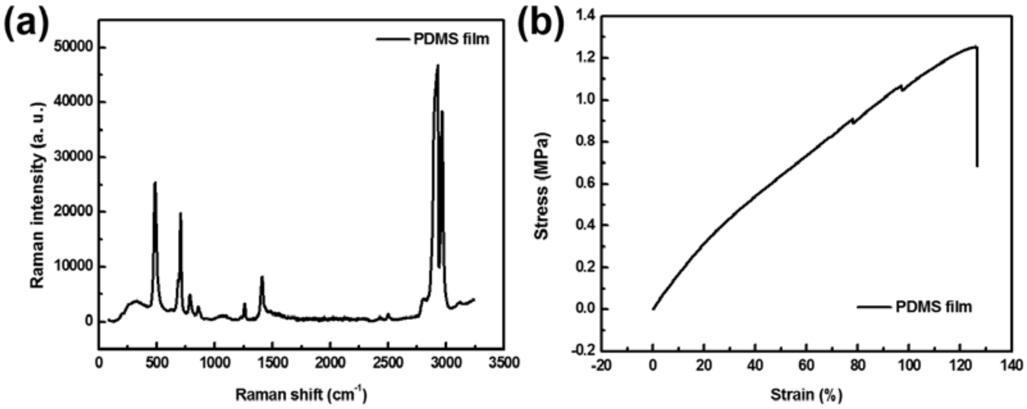

Figure S3. (**a**) Raman spectrum of PDMS film. (**b**) Stress vs. strain curve.
